# Oxygen‐Assisted MOCVD Growth of Monolayer PtSe_2_ Films With Bandgap Opening for Semiconducting FET Channels

**DOI:** 10.1002/advs.76362

**Published:** 2026-06-30

**Authors:** Yuseok Kim, Hee‐Soo So, Minseok Yoo, Saeyoung Oh, Dongyoung Kim, Minseung Gyeon, Min‐Kyung Jo, Gichang Noh, Tae Soo Kim, Min‐gyu Kim, Jeongwon Park, Hyun‐Jun Chai, Minsoo Kang, Suhyun Kim, Ayoung Ham, Jaehyun Lee, Jongsun Lim, Seungwoo Song, Joon Young Kwak, Seunghwan Seo, Chang‐Soo Lee, Chang Gyoun Kim, Kibum Kang

**Affiliations:** ^1^ Department of Materials Science and Engineering Korea Advanced Institute of Science and Technology (KAIST) Daejeon Republic of Korea; ^2^ Thin Film Materials Research Center Korea Research Institute of Chemical Technology (KRICT) Daejeon Republic of Korea; ^3^ 2D Device Technical Unit Samsung Advanced Institute of Technology Suwon Republic of Korea; ^4^ Graduate School of Semiconductor Technology Korea Advanced Institute of Science and Technology (KAIST) Daejeon Republic of Korea; ^5^ Operando Methodology and Measurement Team, Interdisciplinary Materials Measurement Institute Korea Research Institute of Standards and Science (KRISS) Daejeon Republic of Korea; ^6^ Division of Electronic and Semiconductor Engineering Ewha Womans University Seoul Republic of Korea

**Keywords:** field‐effect transistor (FET), MOCVD, monolayer, PtSe_2_, semiconducting bandgap

## Abstract

As device miniaturization approaches its physical limits, the performance enhancement of silicon electronics has become increasingly difficult, shifting attention toward 2D semiconductors as potential alternatives. Among these, monolayer platinum diselenide (PtSe_2_) has garnered significant interest as a next‐generation channel material for nanoelectronics. Distinguished by its exceptional theoretical carrier mobility—six times higher than that of MoS_2—_and remarkable air stability, monolayer PtSe_2_ emerges as a promising candidate for advanced semiconductor applications. However, achieving uniform growth of high‐quality monolayer PtSe_2_ presents challenges. In this study, we report the first successful growth of high‐quality monolayer PtSe_2_ films using an optimized metal‐organic chemical vapor deposition (MOCVD) process. We confirmed the uniform growth of the monolayer films over an area of 1.5 cm × 1.5 cm through various optical analyses, proving superior controllability of precursor flow and growth rate. Oxygen was introduced during the growth process to effectively eliminate carbon impurities, resulting in a high‐quality film. Finally, we demonstrated an array‐level transistor employing the monolayer PtSe_2_ as the channel, achieving low off‐current and a maximum *I*_ON_/*I*_OFF_ ratio of 8.31 × 10^4^. We succeeded in growing an industrially applicable level of semiconducting PtSe_2_ film, thereby highlighting the advantages of our growth method for future electronic applications.

## Graphical abstract

Monolayer PtSe_2_ films are successfully grown via optimized MOCVD, achieving uniform coverage over a 1.5 cm × 1.5 cm area. Oxygen‐assisted growth effectively removes carbon impurities, ensuring high film quality. Array‐level FETs based on monolayer PtSe_2_ channels demonstrate low off‐current and high I_ON_/I_OFF_ ratios, highlighting the potential of PtSe_2_ for future nanoelectronic applications.

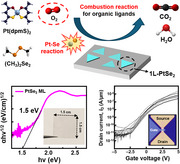

## 1 Introduction

PtSe_2_, a member of the group‐10 2D transition metal dichalcogenides (TMDs) [1, 2], is renowned for its atomically thin structure, exceptional electronic properties, and versatility, making it a promising material for electronic applications [3, 4, 5, 6, 7, 8, 9, 10]. PtSe_2_ exhibits a pronounced bandgap transition, shifting from a semiconducting bandgap of 1.42 eV in the monolayer level to a semi‐metallic state in the multilayer level stemming from its strong interlayer coupling [11]. With this significant variation in electrical conductivity, a single PtSe_2_ material can simultaneously exhibit semiconducting and metallic behaviors within a single device. It also features a theoretical charge carrier mobility of approximately 1892 cm^2^/V∙s even at the monolayer level—nearly six times higher than that of MoS_2_ [1, 12]. Additionally, it stands out among 2D materials due to its exceptional air stability, exhibiting one of the lowest Δ*G* values of oxidation [13]. These outstanding properties have driven extensive research on PtSe_2_ across various fields, especially in nanoelectronics, such as from low‐resistance electrodes to transistor channel materials, and even facilitated the development of all‐PtSe_2_ field‐effect transistors (FETs) [14, 15].

While extensive research has focused on the growth of multilayer PtSe_2_, achieving uniform and high‐crystalline monolayer PtSe_2_ films—despite their semiconducting properties—remains a considerable challenge. Various methods, including thermally assisted conversion (TAC) [4, 16], chemical vapor deposition (CVD) with powder sources [17, 18], and molecular beam epitaxy (MBE) [19, 20], have been utilized to grow monolayer PtSe_2_ films. However, these approaches have typically yielded nonuniform multilayer films with very small grain size (10–20 nm) or multilayer flakes partially composed of very small monolayer regions, falling short in achieving precise thickness control, uniform growth, and successful transistor demonstrations due to the strong interlayer interaction of PtSe_2_ [21]. Driven by the strong demand for monolayer PtSe_2_ with its remarkable semiconducting properties, addressing these issues is crucial for harnessing the full potential of PtSe_2_ as a next‐generation material in advanced electronic applications.

In this work, we demonstrate the successful growth of monolayer PtSe_2_ films using gas‐phase supply growth in an industry‐compatible process [22, 23, 24], confirming their clear semiconducting properties through optical and electrical measurements. The key to this excellent process lies in the novel Pt organic precursor with a high decomposition temperature (∼300°C), which suppresses gas‐phase nucleation, and the new MOCVD chemistry involving oxygen, which effectively eliminates carbon impurities and enables precise control over the growth dynamics. Through various optical analyses, we confirmed the semiconducting bandgap (∼1.5 eV) and outstanding uniformity of the monolayer PtSe_2_ film across the 1.5 cm × 1.5 cm substrate. Furthermore, an array‐level FET was fabricated using the grown monolayer PtSe_2_ film as the channel layer. We achieved a low off‐current and high *I*_ON_/*I*_OFF_ ratio (∼10^5^), attributed to the large bandgap characteristics of the monolayer PtSe_2_, demonstrating the high performance of FETs. Through these results, we demonstrated the successful realization of a uniform monolayer PtSe_2_ film over a large area, with sufficient quality for FET‐grade applications and array‐level device integration—an achievement not previously demonstrated in earlier studies. Our findings establish PtSe_2_ as a promising FET channel candidate that pairs excellent monolayer‐level electronic performance with compatibility with industrial MOCVD processes, presenting a new paradigm for future nanoelectronics.

## 2 Results and Discussion

### 2.1 Growth of Uniform Monolayer PtSe_2_ via MOCVD

**FIGURE 1:**
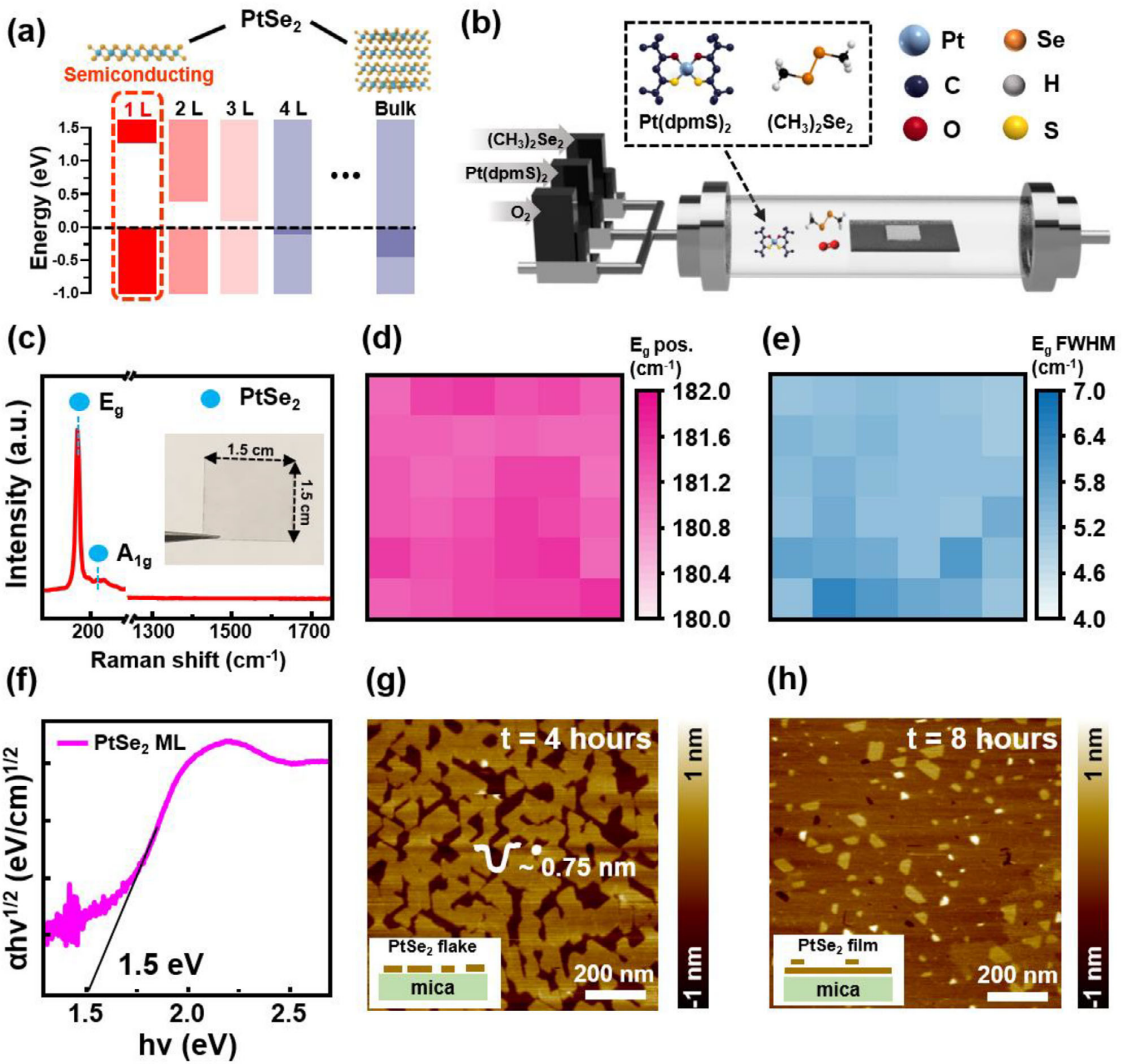
Growth of uniform monolayer PtSe_2_ film via MOCVD. (a) Schematic illustration of the tunable bandgap of PtSe_2_ as a function of layer number, highlighting the semiconducting nature of monolayer PtSe_2_. (b) Schematic illustration of the MOCVD system designed for the growth of monolayer PtSe_2_ film, where Pt(dpmS)_2_ and (CH_3_)_2_Se_2_ are used as precursors. (c) Raman spectrum of monolayer PtSe_2_ film and photograph of PtSe_2_ film grown on 1.5 cm × 1.5 cm muscovite mica substrate (inset). (d, e) Spatial heatmaps of the Raman position (d) and FWHM (e) of the E_g_ peak, analyzed across 36 different regions on the PtSe_2_ film shown in (c). (f) Tauc plot of (αhν)^1/2^ vs hν obtained through Ultraviolet‐visible (UV–vis) analysis. (g, h) AFM mapping images and height profiles of PtSe_2_ film after growth for 4 h (g) and 8 h (h), respectively.

Figure 1 presents an overview of the monolayer PtSe_2_ films grown via our optimized MOCVD process. Figure 1a shows a schematic illustration of the bandgap evolution in PtSe_2_, which varies with the number of layers. The bandgap of monolayer PtSe_2_ is approximately 1.2–1.5 eV [12, 25], and it decreases significantly with the addition of further layers. Specifically, the bandgap drops to ∼ 0.3 eV in the bilayer and falls below 0.1 eV in the trilayer. Beyond four layers, the bandgap drops to 0 eV, and the material becomes semi‐metallic [26]. This drastic shift in the electronic structure emphasizes the importance of monolayer film growth to achieve the desired semiconducting channel characteristics in PtSe_2_. Figure 1b shows the schematic of the MOCVD system we designed for the growth of a monolayer PtSe_2_ film. In this process, Pt(dipivaloylmethane‐sulfonate)_2_ (Pt(dpmS)_2_) and (CH_3_)_2_Se_2_ were used as precursors with N_2_ as the carrier gas. Pt(dpmS)_2_ was specifically synthesized to exhibit superior thermal stability, with a decomposition temperature of approximately 300°C, higher than that of any other Pt precursor (detailed information provided in Figure S1) [27]. This property minimizes the formation of highly reactive radicals under the high temperature conditions of MOCVD, thereby suppressing gas‐phase reactions, which can cause uncontrolled nucleation and defects in the film [28, 29]. Figure 1c shows the representative Raman spectrum of the monolayer PtSe_2_ film, and the inset shows the photograph of the film [5]. The careful design of a new precursor improved growth control, leading to the successful growth of monolayer PtSe_2_ films over a large area of 1.5 cm × 1.5 cm scale on muscovite mica substrates at a growth temperature of 470°C.

Figure 1d,e present spatial heatmaps of the Raman peak position and full width at half maximum (FWHM), corresponding to the E_g_ vibrational mode, obtained from 36 different points on the film shown in the inset of Figure 1c. The E_g_ peak represents the in‐plane vibrational mode of the PtSe_2_ lattice and shifts depending on the number of layers [30]. For monolayer PtSe_2_, the E_g_ peak is observed at ∼181.4 cm^−1^, shifting to lower frequencies as the number of layers increases (Figure S4) [19, 31, 32]. The average E_g_ peak position of the grown film (Figure 1d) was measured at 181.35 cm^−1^, consistent with previous reports [19, 31, 32], with a standard deviation of 0.16 cm^−1^, indicating uniform monolayer growth across the substrate. The average FWHM of the E_g_ peak (Figure 1e) was 5.42 cm^−1^, with a standard deviation of 0.27 cm^−1^. The narrow FWHM suggests a low density of point defects and minimal lattice distortions, confirming the high crystallinity and layer uniformity over a large area of the monolayer PtSe_2_ film [19, 20, 32, 33].

UV–vis measurements were conducted to confirm the optical bandgap of the monolayer PtSe_2_ film (Figure 1f). The absorption coefficient (α) was calculated as $\alpha =\frac{2.303\cdot A}{t}\;$ (A: absorbance, t: thickness) (see Section 4 and Figure S5 for detailed calculation). Given that PtSe_2_ has an indirect bandgap, a Tauc plot was constructed by plotting αhν^1/2^against hν, which revealed an optical bandgap of approximately 1.5 eV, consistent with theoretical predictions [25]. These results verify the large‐area growth of the semiconducting monolayer PtSe_2_ film.

High‐resolution atomic force microscopy (AFM) images (Figure 1g,h) further demonstrate the layer‐by‐layer growth mode of the MOCVD‐grown PtSe_2_. After 4 h of growth, distinct triangular PtSe_2_ grains were observed (Figure 1g). The step height of the partially covered PtSe_2_ film is 0.75 nm, corresponding to the monolayer thickness [6]. The uniformly distributed PtSe_2_ grains, with an average lateral size of approximately 100 nm, suggest simultaneous nucleation followed by lateral growth. Notably, the triangular flakes show preferential orientations along the six‐fold symmetry of the mica substrate, indicating the potential for single‐crystalline film growth (Figure S6) [34, 35, 36].

Extending the growth time to 8 h resulted in the achievement of a fully covered monolayer film, as shown in Figure 1h. The presence of some bilayer flakes is attributed to the stronger interlayer interactions in PtSe_2_ compared to typical TMDs like MoS_2_, leading to overgrowth before complete formation of the monolayer [5]. By employing precise control over precursor flow and lowering growth rate, we successfully minimized the formation of bilayer flakes, achieving a highly uniform monolayer film over a large area—an accomplishment not previously reported (Table S1) [4, 5, 14, 16, 17, 18, 19, 20, 37, 38, 39, 40, 57, 58, 59, 60]. Previous research faced challenges in controlling the number of layers, yielding nonuniform multilayer flakes or films with only small partial monolayer regions. In contrast, our approach achieves high monolayer uniformity over an area of 1.5 cm × 1.5 cm, making it viable for array‐level FET channel applications.

Bilayer PtSe_2_ film growth was also successfully achieved through layer‐by‐layer growth, demonstrating our high layer controllability and film uniformity in a large area (Figure S7). X‐ray diffraction (XRD) analysis confirmed the layered arrangement of the bilayer film, indicated by the detection of the (001) peak of PtSe_2_, which is absent in the monolayer film (Figure S8) [14].

### 2.2 Atomic‐Scale Structural Characterization of Monolayer PtSe_2_ Films

**FIGURE 2:**
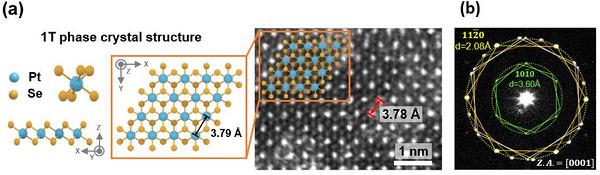
Atomic‐scale structural characterization of monolayer PtSe_2_ films. (a) Schematics of 1T‐phase atomic structure (side view and top view) and HAADF‐STEM image of PtSe_2_ viewed from the [001] direction. (b) SAED pattern of the corresponding sample.

Atomic‐scale structural characterization of the monolayer PtSe_2_ film was performed using a high‐angle annular dark‐field scanning transmission electron microscope (HAADF‐STEM), as shown in Figure 2. Figure 2a provides schematic representations of the atomic structure of the 1T‐phase PtSe_2_ from both side and top views, along with a top‐view HAADF‐STEM image. The intensity of elements in HAADF‐STEM is proportional to Z^α^ (Z is an atomic number, α = 1.5∼2), allowing for the distinction between the bright platinum atoms (Z = 78) and the relatively dimmer selenium atoms (Z = 34) [40]. Each Pt atom is coordinated with six Se atoms, and all three atomic layers (Se‐Pt‐Se) are clearly visible in the top view without any overlap, confirming the 1T crystal structure (Figure 2b and Figure S9) [18]. The lattice constant of our monolayer PtSe_2_ was measured as 3.78 Å, closely matching the theoretical value of 3.79 Å [26]. Figure 2b shows the selected area electron diffraction (SAED) pattern of the PtSe_2_ film, taken along the [001] zone axis, covering a region approximately 2.8 µm in diameter. Diffraction spots corresponding to the {101̅0} and {112̅0} planes are prominently visible. Among these, two sets of six discrete spots in a hexagonal orientation, corresponding to {101̅0} and {112̅0} planes each, appear slightly brighter, indicating that the grains show preferential orientation with alignment to the mica substrate [41, 42]. While most of the grains are aligned along the hexagonal orientation of the mica substrate, the film is not fully epitaxial. As a result, some misaligned grains exist, which lead to the appearance of multiple sets—each consisting of six discrete spots in a hexagonal arrangement—of diffraction spots corresponding to the {101̅0} planes and {112̅0} planes in the SAED pattern.

### 2.3 Effects of Oxygen on High‐Quality PtSe_2_ Film Growth

**FIGURE 3:**
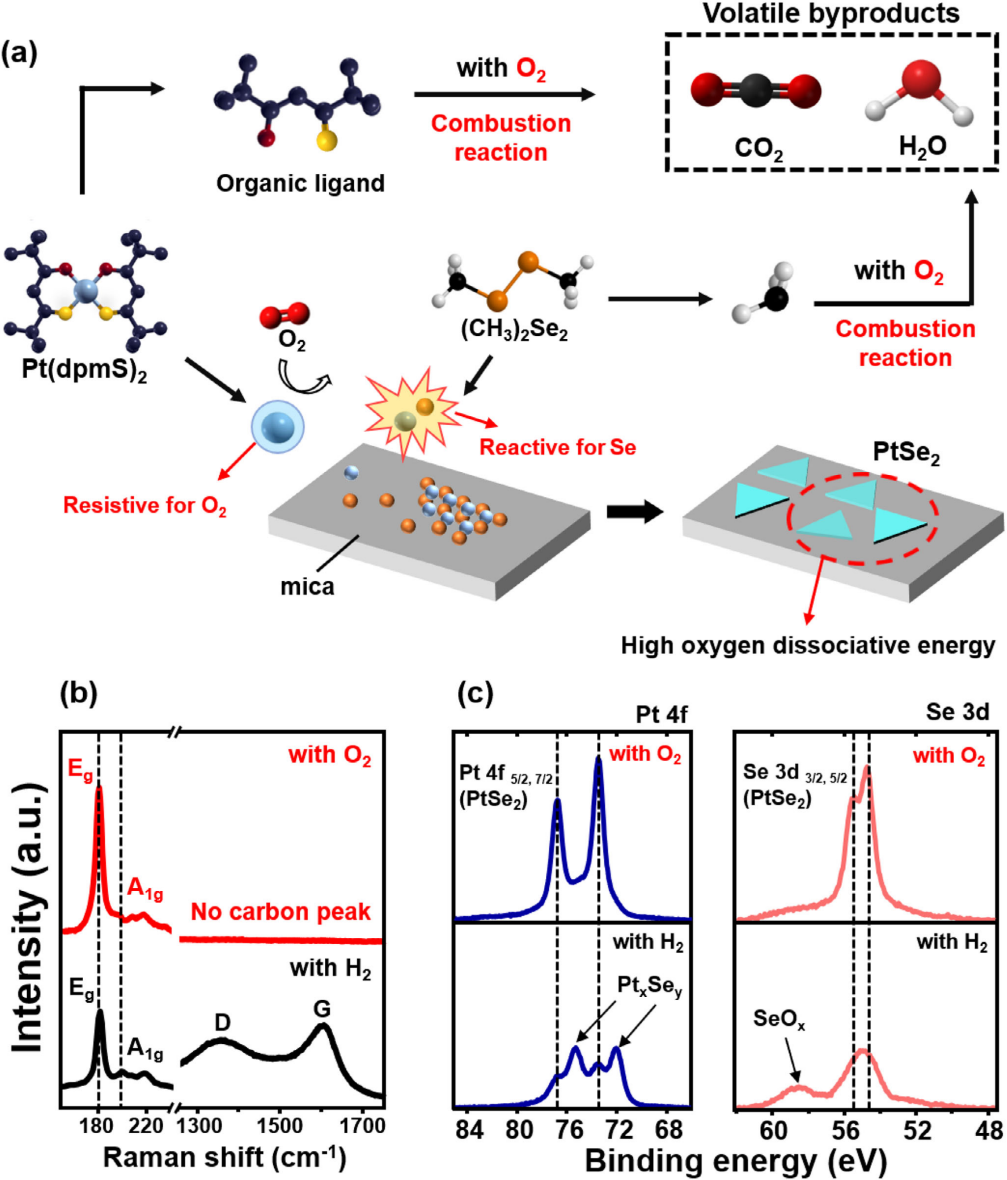
Effects of oxygen on high‐quality PtSe_2_ film growth. (a) Schematic representation of the MOCVD growth mechanism utilizing oxygen to eliminate organic ligands. (b) Raman spectra comparing PtSe_2_ films grown with oxygen (upper panel) and those grown with hydrogen (lower panel). (c) XPS spectra showing the Pt 4f and Se 3d core level regions of PtSe_2_ films grown with oxygen (upper panel) and with hydrogen (lower panel).

Figure 3 illustrates the process of enhancing film quality by utilizing oxygen during growth to eliminate carbon impurities. Metal‐organic precursors often lead to the incorporation of organic ligands within the film, degrading its intrinsic properties [43]. While typical TMDs, such as MoS_2_, are vulnerable to oxidation, novel metal dichalcogenides like PtSe_2_ have a high energy barrier for oxygen dissociation, making them inherently resistant to oxidation [5]. This intrinsic property allows for the formation of carbon‐free PtSe_2_ in the presence of oxygen. Figure 3a presents a schematic of the MOCVD process, highlighting how oxygen aids in removing organic ligands from the precursor. During the thermal decomposition of Pt(dpmS)_2_ and (CH_3_)_2_Se_2_, Pt and Se atoms are released alongside organic ligands. If these ligands remain on the substrate, they can serve as undesirable nucleation sites, resulting in small grain size and induce carbon contaminations that can negatively affect electrical properties [43, 44, 45, 46]. The co‐injection of oxygen triggers a combustion reaction that converts organic ligands into volatile byproducts, such as CO_2_ and H_2_O, while Pt and PtSe_2_ remain unaffected by the oxygen [47]. Consequently, by selectively removing the ligands without damaging PtSe_2_, high‐quality monolayer PtSe_2_ films free of carbon impurities can be produced through oxygen‐assisted MOCVD. To evaluate the air stability of the PtSe_2_ film, we measured the Raman spectra, specifically focusing on the E_g_​ peak position and FWHM, before and after 7 months, as presented in Figure S10. As anticipated, the as‐grown PtSe_2_ film exhibited excellent air stability, with changes in the Raman peak position and FWHM limited to less than 2.21 x 10^−4^% and 2.56%, respectively.

Figure 3b compares the Raman spectra of films grown with and without oxygen. The oxygen‐assisted PtSe_2_ film (red) shows no signals in the range from 1300 to 1600 cm^−1^, whereas the hydrogen‐assisted PtSe_2_ film (black) exhibits D‐band and G‐band Raman peaks, indicating the presence of amorphous carbon (detailed data information provided in, Figure S11) [43]. This finding highlights the critical role of oxygen in removing carbon impurities during the growth process. Additionally, X‐ray photoelectron spectroscopy (XPS) spectra (Figure 3c) further confirm the higher degree of chemical purity and crystallinity in the oxygen‐assisted PtSe_2_ films. The films grown with oxygen (top) display sharper and more symmetric peaks in both the platinum 4f and selenium 3d regions. In contrast, the films grown with hydrogen (bottom) show a sub‐stoichiometric phase (Pt_x_Se_y_) in the Pt 4f core level and SeO_x_ signals in the Se 3d core level [48]. The presence of remaining organic ligands interferes with the bonding of Pt and Se atoms, resulting in deviation from stoichiometry and defects in PtSe_2_ film, while unreacted Se is oxidized to SeO_x_ in air [48, 49]. In conclusion, the incorporation of oxygen during growth significantly reduces defects and facilitates the production of high‐quality films, as evidenced by the aforementioned results.

### 2.4 Monolayer PtSe_2_ FET Array

**FIGURE 4:**
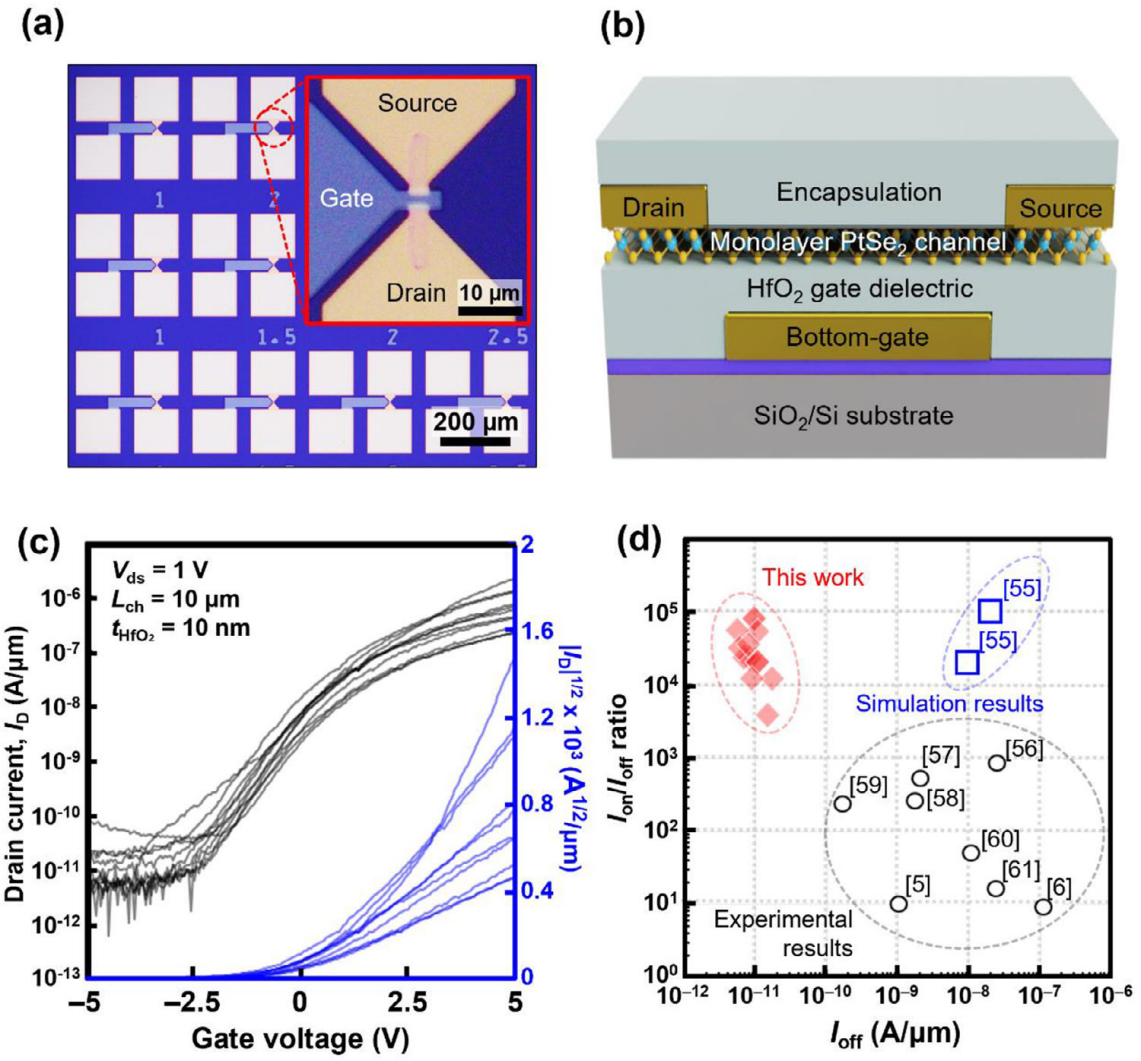
Electrical properties of an array‐level FET with monolayer PtSe_2_ channels. (a) Optical microscopy images of the fabricated monolayer PtSe_2_ FET array and a single PtSe_2_ FET (inset), respectively. (b) Schematic illustration showing the structure of the monolayer PtSe_2_ FET. (c) Transfer characteristic curves of monolayer PtSe_2_ FETs. (d) Benchmarking of *I*_ON_/*I*_OFF_ ratio with respect to *I*_OFF_ in reported PtSe_2_ FETs.

In Figure 4, we evaluated the electrical characteristics of the MOCVD‐grown monolayer PtSe_2_ semiconductor film. Figure 4a shows an array‐level bottom‐gate FET fabricated on the substrate, utilizing monolayer PtSe_2_ as the channel layer. The inset in Figure 4a provides an enlarged optical microscopy (OM) image of a single PtSe_2_ FET device. The channel length (L_ch_) and width (W_ch_) are 1 and 3 µm, respectively. These devices were fabricated using a standard photolithography process, demonstrating the applicability of our growth method at the device level. While the current device dimensions are larger than those in advanced Silicon‐based semiconductor technology, further miniaturization is expected to be achievable through the adoption of high‐resolution lithographic techniques such as electron‐beam lithography or extreme ultraviolet lithography. Figure 4b presents a schematic of the PtSe_2_ FET structure. The FET features local bottom‐gate electrodes and a 10 nm‐thick HfO_2_ gate dielectric deposited on the electrodes. The monolayer PtSe_2_ film was subsequently transferred onto the gate/dielectric‐patterned substrate, followed by the deposition of drain/source metal contacts and a HfO_2_/Al_2_O_3_ encapsulation layer (details provided in Section 4). Next, as depicted in Figure 4c, we investigated the electrical characteristics of the fabricated PtSe_2_ FET array by measuring the transfer characteristics of the devices. Drain‐source voltage (V_DS_) of 1 V was applied, and the resulting transfer curves show a maximum *I*_ON_/*I*_OFF_ ratio of 8.31 × 10^4^ and a minimum *I*_OFF_ of 3.40 × 10–^12^ A/µm with average values of 2.67 × 10^4^ (for *I*_ON_/*I*_OFF_ ratio) and 8.86 × 10–^12^ A/µm (for *I*_OFF_), respectively. In addition, we verified the field‐effect mobility with a value of up to 1.37 cm^2^/V∙s, which was estimated using the transconductance peak (detailed calculation provided in Section 4).

The relatively low field‐effect mobility observed in our PtSe_2_ FETs can be understood by distinguishing intrinsic channel limitations from extrinsic, fabrication‐related effects. From an intrinsic perspective, the MOCVD‐grown PtSe_2_ films exhibit a relatively small grain size (∼100 nm), as confirmed by AFM (Figure 1g). This results in a high density of grain boundaries, which act as scattering centers and limit carrier transport within the channel [50]. From an extrinsic standpoint, several fabrication‐related factors further degrade device performance. First, the PtSe_2_ films are transferred from mica substrates, which can introduce transfer‐related residues and interfacial contamination. Second, the chemical inertness of PtSe_2 —_while beneficial for air stability and reliability—makes channel patterning more challenging, possibly leading to photoresist residue and subsequent degradation of channel quality. Third, interface traps at the HfO_2_ dielectric can induce additional carrier scattering and charge trapping [51, 52, 53, 54]. Finally, the choice of contact metal has not yet been fully optimized, potentially leading to non‐ideal carrier injection at the PtSe_2–_metal interface.

As shown in Figure 4d, we additionally compared the extracted *I*_ON_/*I*_OFF_ ratio and *I*_OFF_ values of our device with those reported for previous PtSe_2_ FETs measured at room temperature [5, 6, 55, 56, 57, 58, 59, 60, 61]. Our transistors show the record‐high performance, exhibiting the highest *I*_ON_/*I*_OFF_ ratio and the lowest *I*_OFF_ current among previously reported PtSe_2_ film‐based transistors. This improvement is attributed to the large bandgap of the monolayer PtSe_2_ film achieved through our approach. The increased bandgap effectively suppresses thermally activated carriers in the off state, thereby reducing the off‐current [62]. In contrast, previously reported few‐layer or multilayer PtSe_2_ FETs often exhibit high off‐currents due to the semi‐metallic nature of thicker films. Although the *I_ON_/I_OFF_* ratio of 2.67 × 10^4^ is slightly lower than that of some MoS_2_‐based FETs, it is the highest among PtSe_2_ film‐based transistors, which typically suffer from high off‐state leakage due to their narrow bandgaps. This unprecedented performance highlights the success of our approach in growing uniform, atomically thin PtSe_2_ films, overcoming challenges in achieving uniform monolayer PtSe_2_ films.

## 3 Conclusion

In summary, we report the first successful growth of uniform large‐scale monolayer PtSe_2_ films using our optimal MOCVD system, which exhibit semiconducting properties. By using Pt(dpmS)_2_, a novel precursor with high thermal stability, we minimized gas‐phase reactions, which can cause uncontrolled nucleation and impurities. Unlike other TMDs that are prone to oxidation, PtSe_2_ possesses strong resistance to oxidation, enabling the effective use of oxygen during the growth process to eliminate carbon impurities. By meticulously controlling the flow of Pt(dpmS)_2_ and oxygen, we achieved high‐quality monolayer PtSe_2_ films on muscovite mica substrates. The monolayer films demonstrated high optical uniformity over a large area (1.5 cm × 1.5 cm) and an optical bandgap of approximately 1.5 eV. In addition, we successfully demonstrated a PtSe_2_ FET array, showcasing the electrical properties of semiconducting PtSe_2_. Notably, we achieved the highest *I*_ON_/*I*_OFF_ ratio and the lowest *I*_OFF_ among bottom‐up synthesis methods reported to date, with a maximum *I*_ON_/*I*_OFF_ ratio of 8.31 × 10^4^ and a minimum *I*_OFF_ of 3.40 × 10–^12^ A/µm. We present a methodology for the controlled growth of group‐10 TMD films by designing an MOCVD system with a novel combination of precursors, enabling the first demonstration of large‐area growth of semiconducting monolayer PtSe_2_. This approach not only establishes PtSe_2_ as a viable material for nanoelectronics but also provides a foundational framework for developing scalable MOCVD growth strategies for other emerging 2D semiconductors. Future research should focus on optimizing growth conditions to enhance grain size and metal contacts for higher FET performance, as well as exploring diverse device applications, paving the way for advancements in next‐generation nanoelectronics.

## 4 Experimental Section

### 4.1 MOCVD Growth of PtSe_2_

The monolayer PtSe_2_ films were synthesized using a custom‐built MOCVD system equipped with a 2‐inch diameter horizontal quartz tube. For the growth of monolayer PtSe_2_ films, freshly cleaved muscovite mica substrates, measuring 1.5 cm × 1.5 cm, were employed. The platinum and selenium precursors were Pt(dpmS)_2_, represented as Pt(C_11_H_19_OS)_2_, and (CH_3_)_2_Se_2_, respectively. Nitrogen served as the carrier gas, while oxygen was injected to facilitate ligand removal and ensure layer‐by‐layer growth. All precursors and gases were precisely controlled by mass flow controllers (MFCs). Due to the relatively low vapor pressure of Pt(dpmS)_2_, the canister containing this precursor was heated to 120°C using heating tape, and all gas lines from the canister to the chamber inlet were maintained at 120°C to prevent precursor condensation within the gas lines. (CH_3_)_2_Se_2_ was stored in a bubbler maintained at a constant pressure of 800 Torr and was heated with heating tape to increase its vapor pressure. Both Pt(dpmS)_2_ and (CH_3_)_2_Se_2_ were transported by nitrogen carrier gas. The optimized precursor flow rates were 120 sccm for Pt(dpmS)_2_, 10 sccm for (CH_3_)_2_Se_2_, 0.3 sccm for oxygen, and 380 sccm for nitrogen. A single‐zone furnace was employed, and the growth temperature was set at 470°C. Potassium tert‐butoxide was used as an alkali metal assistant.

### 4.2 Material Characterization

The optical properties of PtSe_2_ were characterized using confocal Raman spectroscopy (Horiba, ARAMIS) with an Ar‐ion continuous wave laser operating at a wavelength of 514.5 nm. The system was calibrated using the silicon peak at 521 cm^−1^. AFM imaging of PtSe_2_ was performed in contact mode AFM (Park systems, NX‐10). For UV–vis characterization, the film was transferred onto a transparent fused silica substrate, and then we obtained the transmittance (T) and reflectance (R) over the 200–2000 nm wavelength range, using a UV–vis/NIR Spectrophotometer (Lambda 1050, PerkinElmer). Total transmittance (T_tot_) and total reflectance (R_tot_) were calculated by subtracting the T and R values of the pristine fused silica substrate from the T and R values of the monolayer PtSe_2_ sample. The absorbance (A) of the film was calculated using the formula by $A=2+log\frac{1}{R_{\mathrm{tot}}+T_{\mathrm{tot}}}$, and the absorption coefficient (α) was determined as $\alpha =\frac{2.303\cdot A}{t}\;$ (t: thickness). Given that PtSe_2_ has an indirect bandgap, the Tauc plot was constructed by plotting αhν^1/2^against hν. For STEM imaging, the PtSe_2_ films were delaminated onto deionized water and subsequently transferred onto Cu TEM grids using a wet‐transfer method. Top‐view STEM images and SAED patterns were acquired using a FEI Titan microscope operated at 200 kV.

### 4.3 PMMA‐Based Wet Transfer Method

First, the edge of the PtSe_2_ film on the mica substrate was removed using a razor blade. Polymethyl methacrylate (PMMA) was spin‐coated onto a clean PDMS surface at the rate of 4000 rpm for 30 s. After the spin‐coating, the PtSe_2_ sample was placed upside down on the PMMA‐coated PDMS surface. The sample was left to dry for 2 h to allow the PMMA to solidify. Subsequently, the sample/PMMA/PDMS stack was dipped into deionized (DI) water, separating the PMMA holding the PtSe_2_ film from the mica substrate. After drying, the PtSe_2_ film was transferred onto the FET‐patterned substrate at 100°C. To enhance adhesion between the film and substrate, vertical pressure was applied to the PDMS for 10 min. The PDMS was then removed from the film/PMMA by increasing the substrate temperature to 190°C. Finally, the substrate was immersed in acetone for 24 h to dissolve and remove the PMMA layer.

### 4.4 Fabrication of PtSe_2_ FET Array

To fabricate the bottom‐gate FET with PtSe_2_, we used a pre‐patterned bottom‐gate SiO_2_ (100 nm)/Si (p^++^) substrate. Titanium (Ti) and gold (Au) were sequentially deposited as gate metal electrodes with thicknesses of 5 and 20 nm, respectively. HfO_2_ was deposited as a gate dielectric using ALD at 200°C (Figure S12). The PtSe_2_ film was then transferred onto the pre‐patterned bottom‐gate substrate, and then top‐contact source and drain electrodes were patterned via photolithography and deposited using an e‐beam evaporator. Gold was chosen as the contact metal. HfO_2_ and Al_2_O_3_ were deposited as encapsulation materials by ALD at 200°C and 150°C, respectively.

### 4.5 Characterization and Parameter Calculation of PtSe_2_ FET

The electrical characterization of PtSe_2_ FET was performed under ambient conditions using a semiconductor parameter analyzer system (Keithley instrument, 4200A‐SCS parameter analyzer). Field‐effect mobility was extracted from the following equation: $\mu_{\mathrm{FET}}=\left(\frac{L}{WC_{\mathrm{ox}}V_{\mathrm{DS}}}\right)\;\times \frac{dI_{\mathrm{DS}}}{dV_{\mathrm{GS}}}$. *L* is the channel length, *W* is the channel width, *C*_ox_ is gate oxide capacitance per unit area, *V*_DS_ is the voltage between source and drain, and *V*_GS_ is the gate voltage. The mobility of PtSe_2_ FET was calculated using the following device parameters: *L* = 0.5∼2.5 µm, *W* = 1∼5 µm, *C*_ox_ = 1 × 10^− 2^ F m^−2^ (*C*_ox_ = *ε*_0_ *ε*_r_/*t*_ox_, *t*_ox_ = 10 nm), and *V*_DS_ = 1 V.

## Author Contributions

Y. Kim, H. So, and M. Yoo contributed equally. The manuscript was written through the contributions of all authors. All authors have given approval to the final version of the manuscript.

## Conflicts of Interest

The authors declare no conflicts of interest.

## Supporting information




**Supporting File**: advs76362‐sup‐0001‐SuppMat.docx.

## Data Availability

The data that support the findings of this study are available from the corresponding author upon reasonable request.
