# Supplementary material for: Oxygen‐Assisted MOCVD Growth of Monolayer PtSe2 Films With Bandgap Opening for Semiconducting FET Channels
Source: Adv Sci (Weinh). 2026 Jun 30;13(53):e76362. doi: 10.1002/advs.76362 (PMC13336733; doi:10.1002/advs.76362)
Supplement: Supplementary file 1 [file ADVS-13-e76362-s001.docx]

Supporting Information

Oxygen-Assisted MOCVD Growth of Monolayer PtSe_2_ Films with Bandgap Opening for Semiconducting FET Channels

Yuseok Kim, Hee-Soo So, Minseok Yoo, Saeyoung Oh, Dongyoung Kim, Minseung Gyeon, Min-Kyung Jo, Gichang Noh, Tae Soo Kim, Min-gyu Kim, Jeongwon Park, Hyun-Jun Chai, Minsoo Kang, Suhyun Kim, Ayoung Ham, Jaehyun Lee, Jongsun Lim, Seungwoo Song, Joon Young Kwak, Seunghwan Seo^*^, Chang-Soo Lee^*^, Chang Gyoun Kim^*^ and Kibum Kang^*^

**1. Thermal and structural properties of Pt(dpmS)_2_ and (CH_3_)_2_Se_2_.**

To provide further insight into the metal-organic precursors used in this study, we investigated the thermal and structural properties of Pt(dpmS)_2_ and (CH_3_)_2_Se_2_. The Pt(dpmS)_2_ precursor incorporates a dpmS ligand which is thermally stable and sterically bulky, particularly due to the terminal tert-butyl groups. These features can suppress undesired external interactions with the metal center, contributing to the chemical stability of the precursor. Thermogravimetric analysis (TGA) reveals that Pt(dpmS)_2_ begins to decompose around 250–300 °C (Figure S1), which is among the highest decomposition temperatures reported for Pt-based CVD precursors. This result supports that this precursor is thermally stable and well-suited for minimizing gas-phase reactions.

Figure S1. Thermogravimetric analysis curve of Pt(dpmS)_2_

Given that the Pt(dpmS)_2_ precursor contains Pt–S bonds, the possibility of sulfur incorporation into the grown PtSe_2_ films or the formation of sulfur-containing secondary phases must be carefully examined. To address this, Raman spectroscopy and XPS analyses were performed.

Figure S2a shows the Raman spectra of the PtSe_2_ films measured in the range of 100–550 cm^-1^. The characteristic E_g_ (181.4 cm^-1^) and A_1g_ (208.7 cm^-1^) modes of PtSe_2_ are clearly observed, along with a mica substrate peak near ~270 cm^-1^. Notably, no Raman features are detected in the 300–350 cm^-1^ region, where PtS_2_ (E_g_^1^ ~303.1 cm^-1^, A_1g_^2^ ~344.2 cm^-1^) and PtS (B_1g_ ~336.1 cm^-1^) are expected, indicating the absence of sulfur-containing secondary phases.^1^

Figure S2b shows the XPS narrow scan in the S 2p region (159–173 eV). No detectable peaks corresponding to S 2p_3/2_ and S 2p_1/2_ (typically ~162–164 eV) are observed. Instead, only Se-related peaks (Se 3p_3/2_ ~160 eV and Se 3p_1/2_ ~165.5 eV) are present, confirming that sulfur is not incorporated within the detection limit.

Thermodynamically, based on estimations using reported calorimetric data, PtSe_2_ is expected to possess a lower formation Gibbs free energy than PtS_2_ under the actual growth condition of 470 °C (743 K), with estimated values of ΔGf° ≈ −89 kJ/mol for PtSe_2_ and ≈ −80 kJ/mol for PtS_2_. This indicates that PtSe_2_ remains thermodynamically more favorable by approximately 8–10 kJ/mol at the growth temperature. Under the strongly Se-rich conditions of MOCVD growth, the elevated selenium chemical potential (μSe) further shifts the thermodynamic driving force toward PtSe_2_ formation, thereby promoting chalcogen exchange and suppressing sulfur incorporation.

These results collectively confirm that sulfur incorporation and the formation of PtS_2_ or PtS phases are negligible in the synthesized PtSe_2_ films.

Figure S2a-b. (a) Raman spectra of PtSe_2_ (this work) in the 100–550 cm^-1^ range. (b) XPS S 2p and Se 3p region spectra of PtSe_2_ (this work).

(CH_3_)_2_Se_2_ is a symmetric organoselenium molecule consisting of two methyl groups bonded to a linear Se–Se backbone. Due to its chemical reactivity and the instability of its decomposition byproducts, direct thermal analysis is challenging. Instead, we provide a schematic illustration of its molecular structure and the primary decomposition pathways (Figure S3). The molecule undergoes thermolysis via two comparable bond cleavage routes: (1) Se–C bond cleavage, producing methyl radicals (·CH_3_) and selenium-containing fragments, and (2) Se–Se bond cleavage, yielding two ·SeCH_3_ radicals. The respective bond dissociation energies are approximately 198 kJ/mol and 202 kJ/mol, as reported in prior studies.^2, 3^

The relatively low thermal stability of (CH_3_)_2_Se_2_ compared to other selenium precursors such as (CH_3_)_2_Se allows for efficient decomposition below 400 °C, enabling a high selenium chemical potential at our growth temperature of 470 °C. This selenium-rich environment plays a key role in stabilizing the stoichiometric 1T phase of PtSe_2_ and in promoting uniform, high-quality film formation during MOCVD.

Figure S3. Schematic illustration of the possible thermal decomposition pathways of (CH_3_)_2_Se_2_. Two primary dissociation routes are shown: (1) Se–C bond cleavage and (2) Se–Se bond cleavage.

**2. Optical analyses of uniform monolayer PtSe_2_ film**

We successfully achieved a highly uniform monolayer PtSe_2_ film over a 1.5 cm × 1.5 cm area, as confirmed by Raman mapping at 36 different positions and UV-Vis spectroscopy, which indicated a monolayer uniformity and optical bandgap of ~1.5 eV. Raman spectroscopy was employed to analyze the layer-dependent vibrational properties of PtSe_2_. Figure S4 presents the Raman spectra of 1L (monolayer), 2L (bilayer), and Bulk (>8 layers) PtSe_2_ samples, with all spectra normalized to the Si reference peak at 521 cm^-1^. The E_g_ peak exhibits a systematic redshift with increasing layer thickness, shifting from ~181.5 cm^-1^ (monolayer) to ~179.7 cm^-1^ (bilayer) and ~174.9 cm^-1^ (> 8 layers).^4,5,6^ This redshift arises from stacking-induced structural changes and long-range interlayer interactions, which affect the lattice vibrational modes as additional layers are introduced. These observations confirm that Raman peak positions can be reliably used to determine the number of PtSe_2_ layers. Additionally, the A_1g_ peak intensity increases with layer number, but for monolayer PtSe_2_, the A_1g_ peak remains weak, making the E_g_/A_1g_ intensity ratio unreliable for precise layer identification.

To further investigate the optical properties, UV-Vis transmittance and reflectance measurements were conducted. The monolayer PtSe_2_ film was transferred onto a transparent fused silica substrate, and the transmittance (T) and reflectance (R) spectra were collected over the 200–2000 nm wavelength range. The total transmittance (T_tot_) and total reflectance (R_tot_) were obtained by subtracting the values of the pristine fused silica substrate from those of the PtSe_2_-coated sample. As shown in Figure S5a, the transmittance spectrum of PtSe_2_ exhibits a notable reduction in the 300–800 nm range, corresponding to its optical absorption. Similarly, Figure S5b demonstrates an increase in reflectance within the same wavelength range, confirming the light absorption characteristics of PtSe_2_. The absorbance (A) of the film was calculated using the formula by $A=2+log\frac{1}{R_{\mathrm{tot}}{+T}_{\mathrm{tot}}}$, and the absorption coefficient (α) was determined as $\alpha=\frac{2.303\cdot A}{t}$ (t: thickness).

These optical properties highlight the semiconducting nature of monolayer PtSe_2_, further supporting its suitability for nanoelectronic and optoelectronic applications.

**Figure S4.** Raman spectra of monolayer (blue-colored), bilayer (olive-colored), and multi-layer (green-colored) PtSe_2_ films.

**Figure S5**. (a) Transmittance and (b) reflectance of MOCVD-grown monolayer PtSe_2_ film.

**3. Preferential orientation of monolayer PtSe_2_ flakes**
The PtSe_2_ triangular domains exhibit epitaxial growth on the mica substrate, predominantly in two orientations: a majority (highlighted by red circles) aligned in a single direction, and a minority (highlighted by blue circles) aligned in the opposite direction. This distribution suggests symmetry-guided alignment with the hexagonal lattice of the mica substrate.^7^ Statistical analysis shows that approximately 62 % of the domains are oriented within ±3° of either 0° or 60°, corresponding to the crystallographic symmetry directions of mica (Figure S6). These results indicate a strong tendency for epitaxial or quasi-epitaxial alignment, despite the relatively low growth temperature, although the film does not yet exhibit perfect single-crystalline epitaxy.

Figure S6. (a) AFM image of partially grown monolayer PtSe_2_, showing preferential flake orientations near 0° (red) and 60° (blue). (b) Orientation histograms of the discernible triangular PtSe_2_ flakes in (a).

**4. Layer-by-layer growth of PtSe_2_ film**

Using an optimized MOCVD system, we successfully achieved the uniform growth of monolayer PtSe_2_ films. Beyond monolayer formation, we also demonstrate the controlled growth of bilayer PtSe_2_ films, achieving precise layer-by-layer growth. This was accomplished by carefully controlling the precursor flow and growth rate, which promoted adatom migration to flake edge sites, facilitating the transition from isolated flakes to a continuous bilayer film.

Figure S7a-d present the AFM images illustrating the bilayer PtSe_2_ growth process, showing the gradual transition from partially grown PtSe_2_ flakes to a fully merged bilayer film. This demonstrates the high degree of growth control achieved in our MOCVD system. Further structural confirmation is provided by X-ray diffraction (XRD) analysis, as shown in Figure S7. Unlike monolayer PtSe_2_, which lacks a detectable out-of-plane diffraction peak, the bilayer PtSe_2_ film exhibits a distinct (001) XRD peak at ~17.1°, confirming the formation of a well-ordered layered structure.^8^ These results highlight the precise thickness control and high crystallinity of the bilayer PtSe_2_ film, reinforcing the effectiveness of our MOCVD approach in producing high-quality layered PtSe_2_ films.

**Figure S7.** AFM images of MOCVD-grown PtSe_2_ films. Images from left to right show AFM analysis results of (a) partially grown monolayer PtSe_2_, (b) monolayer PtSe_2_, (c) mono-bilayer PtSe_2_, and (d) bilayer PtSe_2_ films.

**Figure S8.** XRD analysis results of MOCVD-grown multilayer PtSe_2_ films on mica. (a) Narrow range (15° to 19°), showing PtSe_2_ (001) peaks for multilayer PtSe_2_ films. (b) Wide range (10° to 45°). The right graph shows a magnified view of the low-intensity region to highlight minor peaks. Gray circles and orange triangles indicate mica substrate and PtSe_2_ peaks, respectively.

**Figure S9.** Line intensity profile of STEM image of MOCVD-grown monolayer PtSe_2_ film.

**Figure S10.** Verification of air-stability on MOCVD-grown monolayer PtSe_2_ films via Raman analysis. Raman (a) E_g_ peak position and (b) E_g_ FWHM before and after exposure to air for 7 months.

**5. Oxygen-assisted growth of monolayer PtSe_2_**

The use of oxygen during MOCVD growth was found to be critical in achieving high-quality monolayer PtSe_2_ films by effectively removing organic ligands from the precursors. Residual carbon impurities from these ligands can act as undesirable nucleation sites, leading to the formation of small and non-uniform grains. As shown in Figure S11a, oxygen-assisted growth results in monolayer PtSe_2_ flakes with an average grain size of ~150 nm and high size uniformity, progressively merging into a continuous film with increased Pt precursor flow rate (Figure S11b).

In contrast, when hydrogen is used during growth (Figure S11c), the grain size is significantly reduced to 20–50 nm, and the flakes become thick and non-uniform, indicating uncontrolled nucleation and poor layer-by-layer growth (Figure S11d). This direct comparison confirms that oxygen plays a crucial role in removing carbon impurities and suppressing unwanted nucleation, thereby enabling precise control over both grain size and layer uniformity. These findings highlight the importance of oxygen in optimizing the MOCVD process for uniform monolayer PtSe_2_ growth.

**Figure S11.** SEM images of PtSe_2_ films grown under different conditions. (a-b) Oxygen-assisted growth with 0.3 sccm O_2_: (a) 100 sccm (low flow rate), (b) 120 sccm Pt(dpmS)_2_ (high flow rate). (c-d) Hydrogen-assisted growth with 30 sccm H_2_: (c) 100 sccm (low flow rate), (d) 130 sccm Pt(dpmS)_2_ (high flow rate).

**Figure S12.** Structural schematic of PtSe_2_ FET.

**6. Growth of monolayer PtSe_2_ on various substrates**

To investigate substrate-dependent growth behavior, PtSe_2_ films were grown on 16 different substrates. As shown in Figure S13, these substrates are categorized into four groups: (i) layered crystalline, (ii) bulk crystalline, (iii) amorphous materials, and (iv) deposited metal films. High-quality monolayer PtSe_2_ films were consistently obtained only on mica. On certain crystalline substrates, such as sapphire, STO, GaN, and ST-cut quartz, monolayer-like PtSe_2_ flakes were observed; however, precise control over the number of layers was not achieved. In contrast, growth on oxide surfaces such as HfO_2_ and on deposited metal films resulted in significantly poorer film quality, characterized by irregular grain sizes and non-uniform coverage.

These variations are attributed to differences in the substrates' ability to attract adatoms during growth. While materials like sapphire and GaN may also exhibit moderate adatom attraction, the presence of surface potassium ions in mica is particularly effective in electrostatically attracting Pt and Se adatoms and suppressing vertical stacking. This promotes lateral growth directly on the substrate surface, rather than on top of existing PtSe_2_ domains, thereby facilitating uniform monolayer formation. Similar potassium-assisted growth behaviors have been reported in previous studies.^9,10^

**Figure S13.** SEM images of PtSe_2_ grown on various substrates, including layered crystalline (mica, h-BN, HOPG, and MoS_2_), bulk crystalline (sapphire, ST-cut quartz, STO (111), GaN, and SiC), amorphous (SiO_2_/Si wafer, HfO_2_ by ALD, TiO_2_ by ALD, Al_2_O_3_ by ALD, and soda-lime glass), and deposited metal film (Au and Pt film).

**Figure S14.** Cross-sectional TEM image of MOCVD-grown monolayer PtSe_2_ film.

**7. Influence of Relative Pt-to-Se Supply and Oxygen Flow on Edge Growth Behavior**

To investigate the factors governing the edge growth behavior of PtSe_2_ domains, we systematically varied the relative Pt-to-Se supply and oxygen flow conditions (Figure S15).

First, the influence of the relative metal-to-chalcogen supply was examined by fixing the (CH_3_)_2_Se_2_ supply (10 sccm at 69 °C) while increasing the Pt(dpmS)_2_ canister heating temperature and carrier flow rate (115 °C / 60 sccm → 119 °C / 120 sccm → 121 °C / 180 sccm) (Figure S15a). Although the exact vapor-phase composition cannot be quantitatively determined due to the unknown vapor pressure of Pt(dpmS)_2_, these conditions correspond to a progressive increase in the effective Pt(dpmS)_2_ supply relative to (CH_3_)_2_Se_2_. As shown in Figure S15a, the domains evolve from relatively sharp triangular edge configurations to larger and more truncated polygonal edge configurations with increasing Pt(dpmS)_2_ supply. This behavior indicates a reduction in edge growth anisotropy, suggesting that the relative growth rates of different edge terminations become more balanced as the system shifts toward a more metal-rich growth condition.^11^

In addition, the influence of oxygen flow on edge growth behavior was examined (Figure S15b). While triangular edge configurations are generally observed at lower oxygen flow rates, increasing the oxygen flow tends to result in slightly more rounded edge features and partial edge etching. However, this trend is not sufficiently consistent to allow a definitive conclusion. This suggests that the effect of oxygen on edge growth behavior is complex and not governed by a single dominant factor. Increasing oxygen flow can simultaneously influence precursor decomposition, effective Pt(dpmS)_2_ supply, ligand removal, and mild edge etching, making it difficult to isolate its direct role in determining edge termination.

**Figure S15.** (a) Representative AFM images showing PtSe_2_ domains grown under increasing Pt(dpmS)_2_ flow rates (Pt: 115 °C / 60 sccm → 119 °C / 120 sccm → 121 °C / 180 sccm). (b) Representative AFM images showing PtSe_2_ domains grown under increasing oxygen flow rates (O_2_: 0.2-0.4 sccm).

**8. Benchmarking of MOCVD-grown monolayer PtSe_2_ and comparison with other TMD FETs**

By employing the MOCVD technique, the most industrially compatible growth method, we achieved the growth of monolayer PtSe_2_ films with a confirmed bandgap opening of ~1.5 eV, highly uniform monolayer film over a 1.5 cm × 1.5 cm area and a high *I_ON_/I_OFF_* ratio (~10^5^) in FET operation. This work represents the first report of a monolayer PtSe_2_ semiconductor thin film that simultaneously satisfies the key requirements for large-area growth and array formation, as summarized in Table S1.

Good

Fair

Poor

| **Growth method** | **Industrial compatibility** | **Monolayer (ML) formation** | **Film uniformity** | **Application** | **Semiconducting behavior (*I_ON_/I_OFF_*)** | **Reference**  **(In Manuscript)** |
| --- | --- | --- | --- | --- | --- | --- |
| TAC | Powder Se source | ML | Not verified | Photocatalyst | - | 4 |
|  | Powder Se source | From ML to 17 layers | Non-uniform film | Kirigami | - | 16 |
|  | Gas phase Se supply | From 3 layers to 6 layers | Non-uniform film | Array level FETs | Semi-metallic (~900) | 14 |
|  | Powder Se source | 5 layers | Not verified | Array level FETs | Semi-metallic (~248) | 57 |
|  | Powder Se source | 5~6 layers  (2.5 to 3 nm) | Not verified | Single FET device | Semi-metallic (~230) | 58 |
|  | Powder Se source | From 2 to 10 layers | Not verified | Single FET device | Semi-metallic  (~50) | 59 |
|  | Powder Se source | 5~10 layers  (2.5 to 5 nm) | Not verified | Single FET device | Semi-metallic  (~16) | 60 |
| Powder CVD | Powder source | From 6 layers to 17 layers | Flakes | Single FET device | Semi-metallic (15~20) | 18 |
|  | Powder source | From 3 layers to 5 layers | Non-uniform film | Single FET device | Semi-metallic (25~40) | 17 |
|  | Powder source | From ML to 2 layers | Flakes | - | - | 37 |
|  | Powder source | From ML to 3 layers | Flakes | HER | - | 38 |
|  | Powder source | From ML to 30 layers | Nano-ribbon | HER | - | 39 |
| MBE | Low yield, UHV | From ML to 22 layers | Non-uniform film | - | - | 19 |
|  | Low yield, UHV | From ML to 6 layers | Non-uniform film | - | - | 20 |
| Exfoliation | Handling with tape | From ML to bulk | Not verified | Single FET device | Semiconducting (~10^3^) | 5 |
| MOCVD | Gas phase supply, high scalability | Bulk | Non-uniform film | - | - | 40 |
| MOCVD | Gas phase supply, high scalability | ML | 1.5 cm × 1.5 cm scale ML film | Array level FETs | Semiconducting (~8ⅹ10^4^) | This work |

**Table S1.** Benchmarking of the industrial compatibility, ML formation, film uniformity, applications and semiconducting behavior of PtSe_2_ flakes or films produced by various methods.

Table S2 compares the device-level performance metrics of representative MOCVD-grown 2D TMD FET. Only studies reporting array-level device statistics for MOCVD-grown TMD channels are included to ensure a meaningful and fair comparison. The key device metrics, including I_ON_/I_OFF_ ratio, off-current, and mobility, are summarized. For consistency, most values are reported as max (mean) values, while off-current is presented as mean when available. In cases where statistical values are not explicitly provided in the original reports, only the available data are listed without further estimation..

| **Material** | **Substrate** | **# of devices**  **(total devices (analyzed devices))** | **I_ON_/I_OFF_**  **(max (mean))** | **I_OFF_ [A/μm]**  **(mean)** | **Mobility [cm^2^/V∙s]**  **(max (mean))** | **Reference** |
| --- | --- | --- | --- | --- | --- | --- |
| MoS_2_ | SiO_2_ | 510 (-) | ~10^6^ (10^4^~10^5^) | ~10^-10^ | ~35.9 (26.3) | [12] |
|  | SiO_2_ | 2976 (-) | ~10^7^ (~10^6^) | ~10^-11^ | 21 (18) | [13] |
| 2L MoS_2_ | SiO_2_ | 600 (-) | 2.6 ⅹ 10^7^  (> 10^4^) | ~ 10^-10^ | 34.3 (17.2) | [14] |
|  | SiO_2_/Si wafer | 20 (12) | 5.5 × 10^7^  (1.4 × 10^7^) | - | 33.1 (18.2) | [15] |
| 2L WSe_2_ | c-sapphire | 300 (-) | > 10^7^ (~10^7^) | ~10^-11^ | 13.27 (7.88) | [16] |
| 4L WSe_2_ | SiO_2_ | 340 (340) | >10^4^ (~10^4^) | ~10^-13^ | 6.9 (3~4) | [17] |
| **1L PtSe_2_** | **Mica** | **480 (12)** | **8.31 ⅹ 10^4^**  **(2.67 ⅹ 10^4^)** | **8.86 ⅹ 10^-12^ (3.40 ⅹ 10^-12^)** | **1.37 (0.24)** | **This work** |

**Table S2.** Benchmark comparison of device-level performance metrics for representative MOCVD-grown 2D TMD field-effect transistors.

**References**

(1)	Cullen, C. P.; Ó Coileáin, C.; McManus, J. B.; Hartwig, O.; McCloskey, D.; Duesberg, G. S.; McEvoy, N. Synthesis and Characterisation of Thin-Film Platinum Disulfide and Platinum Sulfide. Nanoscale 2021, 13, 7403–7411. https://doi.org/10.1039/d0nr06197b.

(2)	Tel'noy, V.I.; Sheiman, M. S. Thermodynamics of organoselenium and organotellurium compounds. *Russian Chemical Reviews* **1995**, *64* (4), 309. 10.1070/RC1995v064n04ABEH000152.

(3)	Meija, J.; Beck, T. L.; Caruso, J. A. Interpretation of alkyl diselenide and selenosulfenate mass spectra. *Journal of the American Society for Mass Spectrometry* **2004**, *15* (9), 1325–1332. https://doi.org/10.1016/j.jasms.2004.05.012.

(4)	Yan, M.; Wang, E.; Zhou, X.; Zhang, G.; Zhang, H.; Zhang, K.; Yao, W.; Lu, N.; Yang, S.; Wu, S.; Yoshikawa, T.; Miyamoto, K.; Okuda, T.; Wu, Y.; Yu, P.; Duan, W.; Zhou, S. High Quality Atomically Thin PtSe_2_ Films Grown by Molecular Beam Epitaxy. *2d Mater* **2017**, *4* (4), 045015. https://doi.org/10.1088/2053-1583/aa8919.

(5)	Prechtl, M.; Parhizkar, S.; Hartwig, O.; Lee, K.; Biba, J.; Stimpel-Lindner, T.; Gity, F.; Schels, A.; Bolten, J.; Suckow, S.; Giesecke, A. L.; Lemme, M. C.; Duesberg, G. S. Hybrid Devices by Selective and Conformal Deposition of PtSe_2_ at Low Temperatures. *Adv Funct Mater* **2021**, *31* (46). https://doi.org/10.1002/adfm.202103936.

(6)	Tharrault, M.; Desgué, E.; Carisetti, D.; Plaçais, B.; Voisin, C.; Legagneux, P.; Baudin, E. Raman Spectroscopy of Monolayer to Bulk PtSe_2_ Exfoliated Crystals. *2d Mater* **2024**, *11* (2). https://doi.org/10.1088/2053-1583/ad1e79.

(7)	Zhu, H.; Nayir, N.; Choudhury, T. H.; Bansal, A.; Huet, B.; Zhang, K.; Puretzky, A. A.; Bachu, S.; York, K.; Mc Knight, T. V.; Trainor, N.; Oberoi, A.; Wang, K.; Das, S.; Makin, R. A.; Durbin, S. M.; Huang, S.; Alem, N.; Crespi, V. H.; Van Duin, A. C. T.; Redwing, J. M. Step engineering for nucleation and domain orientation control in WSe_2_ epitaxy on c-plane sapphire. *Nat. Nanotechnol.* **2023**, *18* (11), 1295–1302. https://doi.org/10.1038/s41565-023-01456-6.

(8)	Gyeon, M.; Seo, J. E.; Oh, S.; Noh, G.; Lee, C.; Choi, M.; Kwon, S.; Kim, T. S.; Jeong, H. Y.; Song, S.; Chang, J.; Kang, K. Wafer-Scale Growth of Ultrauniform 2D PtSe₂ Films with Spatial and Thickness Control through Multi-Step Metal Conversion. ***ACS Nano* 2024, *18*** (50), 33977–33987. https://pubs.acs.org/doi/full/10.1021/acsnano.4c08160

(9)	Franceschi, G.; Kocán, P.; Conti, A.; Brandstetter, S.; Balajka, J.; Sokolović, I.; Valtiner, M.; Mittendorfer, F.; Schmid, M.; Setvín, M.; Diebold, U. Resolving the intrinsic short-range ordering of K^+^ ions on cleaved muscovite mica. *Nat. Commun.* **2023**, *14* (1), 208. https://doi.org/10.1002/cphc.202300545.

(10)	Chang, C. S.; Kim, K. S.; Park, B.-I.; Choi, J.; Kim, H.; Jeong, J.; Barone, M.; Parker, N.; Lee, S.; Zhang, X.; Lu, K.; Suh, J. M.; Kim, J.; Lee, D.; Han, N. M.; Moon, M.; Lee, Y. S.; Kim, D.-H.; Schlom, D. G.; Hong, Y. J.; Kim, J. Remote epitaxial interaction through graphene. *Sci. Adv.* **2023**, *9* (42), eadj5379. 10.1126/sciadv.adj5379.

(11)	van der Zande, A. M.; Huang, P. Y.; Chenet, D. A.; Berkelbach, T. C.; You, Y.; Lee, G.-H.; Heinz, T. F.; Reichman, D. R.; Muller, D. A.; Hone, J. C. Grains and Grain Boundaries in Highly Crystalline Monolayer Molybdenum Disulphide. *Nature Materials* 2013, 12 (6), 554–561. https://doi.org/10.1038/nmat3633

(12)	Zhu, J.; Park, J.-H.; Vitale, S. A.; Ge, W.; Jung, G. S.; Wang, J.; Mohamed, M.; Zhang, T.; Ashok, M.; Xue, M.; Zheng, X.; Wang, Z.; Hansryd, J.; Chandrakasan, A. P.; Kong, J.; Palacios, T. Low-Thermal-Budget Synthesis of Monolayer Molybdenum Disulfide for Silicon Back-End-of-Line Integration on a 200 mm Platform. Nature Nanotechnology 2023, 18, 456–463. https://doi.org/10.1038/s41565-023-01375-6

(13)	Kwon, J.; Seol, M.; Yoo, J.; Ryu, H.; Ko, D.-S.; Lee, M.-H.; Lee, E. K.; Yoo, M. S.; Lee, G.-H.; Shin, H.-J.; Kim, J.; Byun, K.-E. 200-mm-Wafer-Scale Integration of Polycrystalline Molybdenum Disulfide Transistors. Nature Electronics 2024, 7, 356–364. https://doi.org/10.1038/s41928-024-01158-4

(14)	Yoo, M. S.; Jung, A.; Yang, S.; Yoo, J. E.; Lee, E.-K.; Byun, K.-E.; Baik, J.; Yun, D.-J.; Park, J.; Kim, J.; Seol, M. Direct Growth and Manufacturing of Single-Crystalline 2D FETs on 8-Inch Si Wafers. IEEE International Electron Devices Meeting (IEDM); IEEE: 2024. https://doi.org/10.1109/iedm50854.2024.10873573

(15)	Kim, T. S.; Noh, G.; Kwon, S.; Kim, J. Y.; Dhakal, K. P.; Oh, S.; Chai, H.-J.; Park, E.; Kim, I. S.; Lee, E.; Kim, Y.; Lee, J.; Jo, M.-K.; Kang, M.; Park, C.; Kim, J.; Park, J.; Kim, S.; Choi, S.-Y.; Song, S.; Jeong, H. Y.; Kim, J.; Kwak, J. Y.; Kang, K. Diffusion Control on the Van der Waals Surface of Monolayers for Uniform Bi-Layer MoS_2_ Growth. Advanced Functional Materials 2024, 34, 2312365. https://doi.org/10.1002/adfm.202312365

(16)	Ghosh, S.; Sadaf, M. U. K.; Graves, A. R.; Zheng, Y.; Pannone, A.; Ray, S.; Cheng, C.-Y.; Guevara, J.; Redwing, J. M.; Das, S. High-Performance p-Type Bilayer WSe_2_ Field Effect Transistors by Nitric Oxide Doping. Nature Communications 2025, 16, 5649. https://doi.org/10.1038/s41467-025-59684-4

(17)	Pendurthi, R.; Sakib, N. U.; Sadaf, M. U. K.; Zhang, Z.; Sun, Y.; Chen, C.; Jayachandran, D.; Oberoi, A.; Ghosh, S.; Kumari, S.; Stepanoff, S. P.; Somvanshi, D.; Yang, Y.; Redwing, J. M.; Wolfe, D. E.; Das, S. Monolithic Three-Dimensional Integration of Complementary Two-Dimensional Field-Effect Transistors. Nature Nanotechnology 2024, 19, 970–977. https://doi.org/10.1038/s41565-024-01705-2
